# Inter-Multilevel Super-Orthogonal Space–Time Coding Scheme for Reliable ZigBee-Based IoMT Communications

**DOI:** 10.3390/s22072695

**Published:** 2022-03-31

**Authors:** Shang-Chih Ma, Mohammad Alkhaleefah, Yang-Lang Chang, Joon Huang Chuah, Wen-Yen Chang, Chiung-Shen Ku, Meng-Che Wu, Lena Chang

**Affiliations:** 1 Department of Electrical Engineering, National Taipei University of Technology, Taipei 10608, Taiwan; muhai@ntut.edu.tw (M.A.); ylchang@ntut.edu.tw (Y.-L.C.); 101583003@cc.ncu.edu.tw (C.-S.K.); 2 Department of Electrical Engineering, Faculty of Engineering, University of Malaya, Kuala Lumpur 50603, Malaysia; jhchuah@um.edu.my; 3 Department of Natural Resources and Environmental Studies, National Dong Hwa University, Hualien 974301, Taiwan; wychang@gms.ndhu.edu.tw; 4 National Space Organization, National Applied Research Laboratories, Hsinchu 30078, Taiwan; momo@narlabs.org.tw; 5 Department of Communications, Navigation and Control Engineering, National Taiwan Ocean University, Keelung 202301, Taiwan; lenachang@mail.ntou.edu.tw

**Keywords:** channel coding, inter-level dependency codes, Internet of Medical Things (IoMT), multilevel coding technique, super-orthogonal space–time codes, ZigBee

## Abstract

The Internet of Things (IoT) technology has revolutionized the healthcare industry by enabling a new paradigm for healthcare delivery. This paradigm is known as the Internet of Medical Things (IoMT). IoMT devices are typically connected via a wide range of wireless communication technologies, such as Bluetooth, radio-frequency identification (RFID), ZigBee, Wi-Fi, and cellular networks. The ZigBee protocol is considered to be an ideal protocol for IoMT communication due to its low cost, low power usage, easy implementation, and appropriate level of security. However, maintaining ZigBee’s high reliability is a major challenge due to multi-path fading and interference from coexisting wireless networks. This has increased the demand for more efficient channel coding schemes that can achieve a more reliable transmission of vital patient data for ZigBee-based IoMT communications. To meet this demand, a novel coding scheme called inter-multilevel super-orthogonal space–time coding (IM-SOSTC) can be implemented by combining the multilevel coding and set partitioning of super-orthogonal space–time block codes based on the coding gain distance (CGD) criterion. The proposed IM-SOSTC utilizes a technique that provides inter-level dependency between adjacent multilevel coded blocks to facilitate high spectral efficiency, which has been compromised previously by the high coding gain due to the multilevel outer code. In this paper, the performance of IM-SOSTC is compared to other related schemes via a computer simulation that utilizes the quasi-static Rayleigh fading channel. The simulation results show that IM-SOSTC outperforms other related coding schemes and is capable of providing the optimal trade-off between coding gain and spectral efficiency whilst guaranteeing full diversity and low complexity.

## 1. Introduction

The Internet of Medical Things (IoMT) technology has been rapidly transforming healthcare delivery in recent years [1]. The IoMT is a subset of the IoT, which pertains to the use of medical devices and applications that are connected over the Internet in order to provide better healthcare services [2]. The IoMT has the potential to improve the accuracy of diagnosis and treatment, enhance patient monitoring, reduce healthcare delivery time, and lower healthcare costs. The IoMT can also engage and empower patients and their caretakers to enhance the quality and efficiency of self-management [3].

The IoMT is typically enabled by wireless devices, wireless sensor networks (WSNs), gateways, Internet protocols, cloud computing, and analytics engines [4]. Among the wireless communication protocols, IEEE 802.15.4-based ZigBee has recently attracted a lot of attention and has become the most popular IoMT-based healthcare solution due to its low cost and low power consumption [5,6,7,8]. ZigBee’s worldwide standard uses the ISM 2.4 GHz frequency band and has a defined rate of 250 kb/s, which make it best suited for periodic data transmission [9,10]. In some chronic disease cases, such as hypertension and diabetes, IoMT-transmitted data typically comprise periodical signals that have relatively low data rates [11]. Hence, the ZigBee protocol is considered to be the ideal choice for applications in which high data rates are not required.

**Figure 1:**
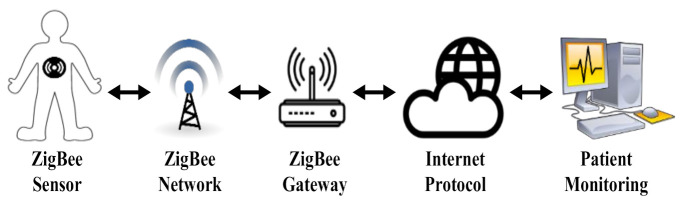
Patient monitoring using a ZigBee wireless network.

The WSN-based IoMT is highly heterogeneous due to various underlying devices, protocols, and applications [12]. Therefore, IoMT systems must be able to appropriately manage numerous devices in terms of interoperability and reliability. The reliability of data that are transmitted in wireless networks is definitely vital to any communication protocol that needs to be ensured. In healthcare systems, the reliability of the communication between ubiquitous IoMT devices is a critical factor [13]. The lack of reliability in IoMT devices may put patients’ lives at risk [14]. Since the ZigBee protocol operates in the license-free 2.4 GHz ISM frequency band that is shared with some other wireless communication protocols, such as IEEE 802.11b/g, there is a potential overlapping or interference that could affect the reliability and robustness of the IEEE 802.15.4 networks [15,16,17,18,19]. Figure 1 shows a remote patient monitoring system that uses a ZigBee wireless network, in which a patient sensor sends periodic data to a ZigBee gateway over a ZigBee network. Then, the data are transmitted over the Internet to a hospital computer to allow medical staff to monitor the patient’s condition.

**Figure 2:**
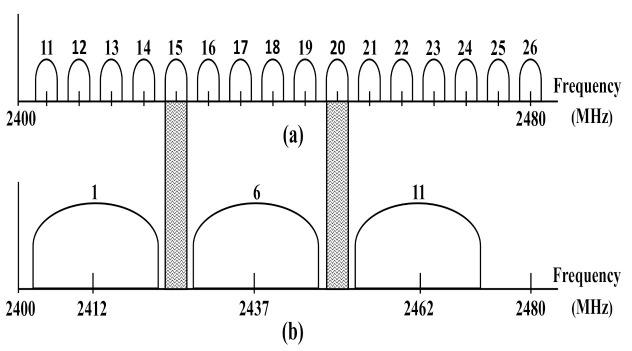
(**a**) IEEE 802.15.4 channels and (**b**) IEEE 802.11b/g non-overlapping channels in North America.

In North America, the IEEE 802.15.4 or ZigBee channels that are located between 802.11b/g channels suffer the most from interference, except for IEEE 802.15.4 channels 15 and 20, which suffer the least from the Wi-Fi or IEEE 802.11b/g interference, as shown in Figure 2. However, IEEE 802.15.4 channels 15, 20, 25, and 26 may not be totally interference-free due to the out-of-band emissions of IEEE 802.11b/g signals.

In recent years, several studies have been conducting to mitigate the coexistence problem of wireless systems, such as interference and overlapping between ZigBee and Wi-Fi channels. Yi et al. [16] proposed a frequency agility-based interference avoidance algorithm for smart grid applications to detect interference and guarantee ZigBee performance when Wi-Fi interference is significant. Kim et al. [5] developed an adaptive algorithm that can control the traffic in Wi-Fi networks to ensure that delays experienced by ZigBee sensors do not exceed the maximum tolerated delay time. Another study [18] proposed a coexistence solution called the guide busy tone (GBT), which can provide reliable communication to the ZigBee network under Wi-Fi traffic influences by reserving a frequency channel for ZigBee. However, the proposed GBT solution requires additional power and cancellation complexity. The work in [19] aimed to design a novel ZigBee decoding technique called PolarScout, which separates collided ZigBee signal samples from Wi-Fi interference to enable direct ZigBee data decoding. Nevertheless, PolarScout imposes restrictions on commodity ZigBee devices.

Yan et al. [20] presented WizBee, a coexistence system that uses a single-antenna sink without changing current Wi-Fi and ZigBee designs. Moreover, an innovative interference cancellation scheme for ZigBee signal coexistence was proposed, in which Wi-Fi decoding is used for channel coefficient estimation in an iterative way. The authors used soft Viterbi decoding with weighted confidence values over interfered subcarriers for robust and accurate Wi-Fi decoding. This work [21] presented a clean-slate technique called DOTA to combat the cross-technology interference (CTI) and enable ZigBee–Wi-Fi co-transmission. Unlike existing solutions that only handle Wi-Fi to ZigBee interference, DOTA accounts for the true sense of CTI wherein ZigBee and Wi-Fi interfere with each other. DOTA comprises two operations: signal polishing and pulse disambiguation to decompose and thread signal samples to recover contaminated ZigBee and Wi-Fi signals. Another work by Yang et al. [22] proposed ZIMO, a sink-based MIMO design on a USRP platform for the harmonic coexistence of ZigBee and Wi-Fi with the aim of protecting the ZigBee data packets from interference by high-power cross-technology signals. The key insight is to properly exploit opportunities that result from the differences between Wi-Fi and ZigBee and to bridge the gap between interested data and cross-technology signals. Additionally, extracting the channel coefficient of Wi-Fi and ZigBee can improve other coexistence technologies.

In this research, a reliable coding scheme is introduced, namely inter-multilevel super-orthogonal space–time coding (IM-SOSTC), which allows for inter-level dependency between adjacent multilevel coded blocks to improve coding gain and guarantee full diversity whilst maintaining reasonable spectral efficiency and decoding complexity in a wireless fading and interfering channel for ZigBee-based IoMT communications. Unlike previous work, this study uses multiple transmitters to further improve the spectral efficiency and achieve full diversity.

## 2. Related Coding Schemes

Many remarkable coding designs have considered low complexity and transmission diversity in wireless fading channels. Alamouti [23] presented a simple transmission diversity scheme to improve signal quality using two transmission antennas and one receiving antenna. The proposed scheme provides the same diversity order as the maximal-ratio receiver combining (MRRC) with one transmission and two receiving antennas. Despite the simplicity of Alamouti’s scheme, there was still a degradation in performance compared to space–time trellis codes (STTCs).

Tarokh et al. [24] designed STTCs to enhance the error performance, provide full diversity gain, and reduce the effects of multi-path fading over wireless communication channels using multiple transmission antennas. Nevertheless, the decoding complexity rises exponentially with the increase in the transmission rate [25]. Another work [26] proposed a simple robust space–time coding scheme using concatenated orthogonal space–time block codes (OSTBC) and trellis coded modulation construction to achieve robust performance over fading conditions. However, the shortcoming of this scheme is that it does not provide the highest possible coding gain. Jafarkhani et al. [27] introduced a new class of STTCs called super-orthogonal space–time trellis codes (SOSTTC), which combine set partitioning (trellis codes) and a super-set of OSTBCs in a systematic way to enhance coding gain and provide full diversity over the previous space-–time trellis code constructions. Although SOSTTC does not expand the constellation signals, it does expand the orthogonal matrices.

Jafarkhani and Hassanpour [28] introduced new codes for four transmission antennas, namely super-quasi-orthogonal space–time trellis codes, which combine set partitioning and a super-set of quasi-orthogonal space–time block codes [29] to improve coding gain and provide full diversity. The authors also presented non-full-rate codes, which provide higher coding gains compared to the full-rate codes. One study [30] proposed a new class of low complexity space–time trellis codes (STTCs) for a closed-loop transmission system according to channel phase feedback and the concatenation of a standard multiple trellis coded modulation (M-TCM) outer code with an inner code, called co-phase STTCs (CPSTTCs). However, this scheme requires a huge overhead for large antenna arrays.

In [31], a multichannel equaliser and orthogonal frequency division multiplexing (OFDM) were applied to the super-orthogonal block codes (SOBCs) in the space–time domain to control the effects of signal interference caused by the frequency selectivity of the fading channel. The authors considered two forms of SOBCs: super-orthogonal space–time trellis coded OFDM and super-orthogonal space–frequency trellis coded OFDM. In addition, new decoding trellises for two-state and four-state coding schemes were introduced. The simulation results showed that the super-orthogonal space–frequency trellis coded OFDM scheme surpassed the super-orthogonal space–time trellis coded OFDM scheme in terms of coding gain under various channel delay spreads. However, the error performance can still be improved.

The multilevel coding (MLC) technique is a powerful approach to construct coding schemes with large coding gains [32,33]. Many previous studies have applied the concatenation of space–time block codes (STBCs) and multilevel codes for the advantages of coding gain and full diversity [27,34,35,36]. However, when multilevel codes schemes utilize the set partitioning of super-orthogonal codes (SOCs), full diversity cannot be achieved, which affects the reliability of ZigBee-based IoMT communications. In this work, a novel technique is proposed, which is called inter-multilevel dependency. This technique provides inter-level dependency between adjacent multilevel coded blocks to provide full diversity and improve the coding gain whilst maintaining reasonable spectral efficiency and decoding complexity for ZigBee-based IoMT communications.

## 3. The Proposed Scheme

**Figure 3:**
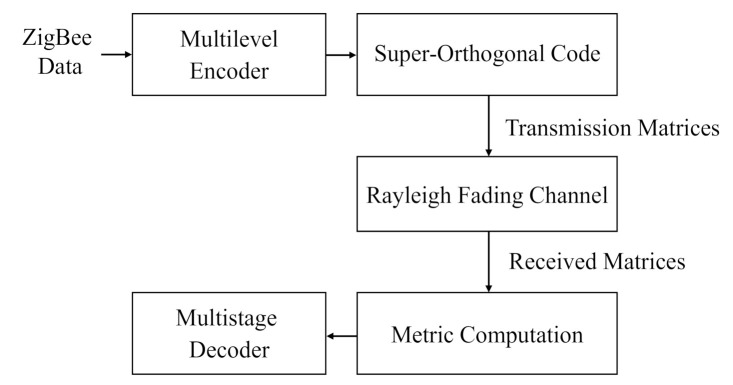
The system flow chart.

In this section, the general design of the proposed system is presented and the set partitioning of a super-orthogonal code is discussed. The superiority of the proposed scheme is demonstrated through the use of examples. Figure 3 shows the overall flow chart for the system model. In the multilevel encoder, each coded block had 2m+1 rows and *n* columns. *m* represents the number of bits per two-dimensional symbol. The *n* columns were mapped to *n* transmission matrices. In the decoder, the squared Euclidean distance was used as the decoding metric and the multistage decoder was adopted.

Starting from the Alamouti 2×2 STBC, a parameterized class of orthogonal transmission matrices for two transmission antennas [27] was defined as follows:

(1) $$T\left(\alpha ,\beta ;\theta \right)=\left(\begin{array}{cc} \alpha \cdot e^{j\theta } & \beta \\ -\beta^{*}\cdot e^{j\theta } & \alpha^{*} \end{array}\right).$$

where α and β are *M*-ary constellation symbols and θ is the rotation angle. During the first time segment, the encoder transmitted $\alpha \cdot e^{j\theta }$ from Antenna 1 and β from Antenna 2. During the second time segment, the encoder transmitted $-\beta^{*}\cdot e^{j\theta }$ from Antenna 1 and $\alpha^{*}$ from Antenna 2.

Many orthogonal space–time block codes (OSTBCs), denoted as $W^{\left(1\right)}\left(\theta \right)=\left\{T(\alpha ,\beta ;\theta )\right\},$ can be built using different values of the parameter θ. The union of these codes is called a super-orthogonal code (SOC) [37]. SOSTTC was designed by concatenating an outer trellis code with an inner super-orthogonal code [38]. The advantage of using super-orthogonal code is that it expands the number of available orthogonal matrices [27,39]. The trellises of SOSTTC were designed using the set partitioning rules to maximize the CGD [40], in which the CGD between two different transmission matrices **T** and **$T^{\prime }$** is defined as:

(2) $$d\left(T,T^{\prime }\right)=\det\left[\left(T-T^{\prime }\right){\left(T-T^{\prime }\right)}^{H}\right].$$

where the superscript *H* denotes the transpose conjugate of any matrix.

**Example** **1.** *Consider the BPSK signal constellation. Let:*

$$\begin{array}{c} W^{\left(1\right)}\left(0\right)=\left\{\left(\begin{array}{cc} +1 & +1\\ -1 & +1 \end{array}\right),\left(\begin{array}{cc} -1 & -1\\ +1 & -1 \end{array}\right),\left(\begin{array}{cc} -1 & +1\\ -1 & -1 \end{array}\right),\left(\begin{array}{cc} +1 & -1\\ +1 & +1 \end{array}\right)\right\} \end{array}$$

*and*

$$\begin{array}{c} W^{\left(1\right)}\left(\pi \right)=\left\{\left(\begin{array}{cc} -1 & +1\\ +1 & +1 \end{array}\right),\left(\begin{array}{cc} +1 & -1\\ -1 & -1 \end{array}\right),\left(\begin{array}{cc} +1 & +1\\ +1 & -1 \end{array}\right),\left(\begin{array}{cc} -1 & -1\\ -1 & +1 \end{array}\right)\right\} \end{array}$$

**Figure 4:**
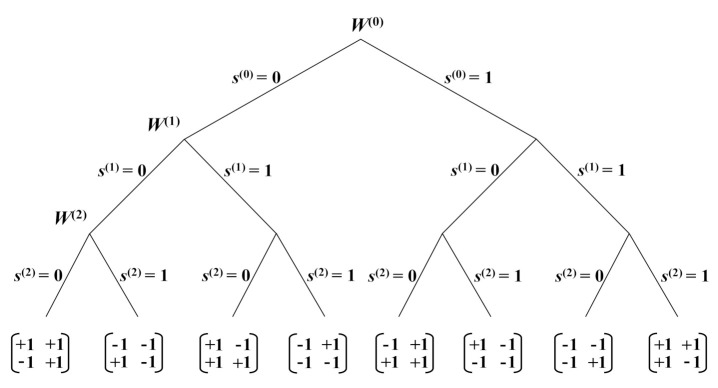
The three-level partition chain in Example 1.

*denote two different OSTBCs with parameter θ=0 and π, respectively. Let $W^{\left(0\right)}$ denote the union of $W^{\left(1\right)}\left(0\right)$ and $W^{\left(1\right)}\left(\pi \right).$ The three-level partitioning chain $P\left(3\right)=W^{\left(0\right)}/W^{\left(1\right)}/W^{\left(2\right)}$ can be formed as indicated in Figure 4. Let the intra-set coding gain distance of $W^{\left(p\right)}{,}_{}p=0,1,2$ be identified as:*

(3) $$\Delta^{\left(p\right)}=\min\left\{d\left(T,T^{\prime }\right)\left|{}_{}T,T^{\prime }\in W^{\left(p\right)}{,}_{}T\neq {T^{\prime }}_{}\right.\right\}.$$

It can be calculated from (2) and (3) that $\Delta^{\left(0\right)},\Delta^{\left(1\right)},$ and $\Delta^{\left(2\right)}$ are 0, 16, and 64, respectively. Note that two different transmission matrices in the super-orthogonal code $W^{\left(0\right)}$ may not be orthogonal. Hence, full diversity cannot be guaranteed in $W^{\left(0\right)}$.

**Figure 5:**
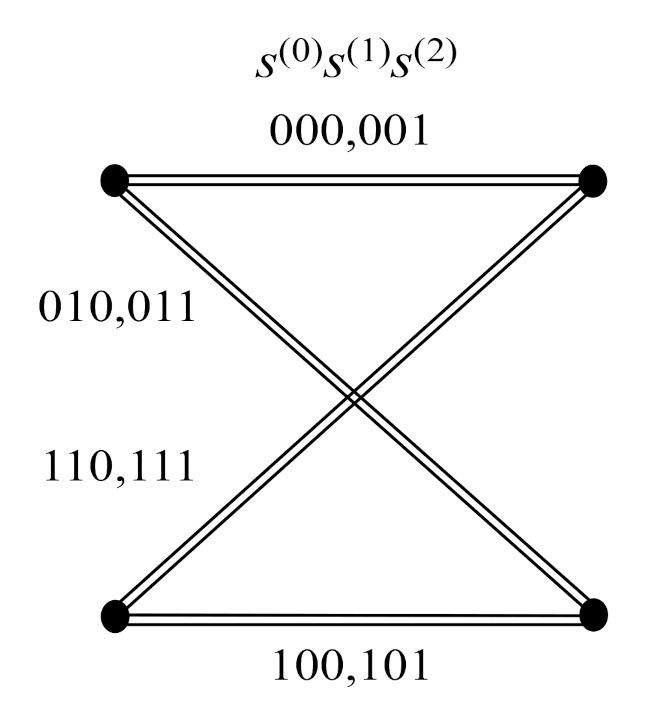
The two-state trellis in Example 1.

A two-state trellis diagram of a 2/3-rate convolutional code encoder is illustrated in Figure 5. Two message bits are encoded into three coded bits, which are then mapped onto one transmission matrix according to the partition chain P(3). Since each transmission matrix contains two BPSK symbols within two time segments, the average spectral efficiency is 1 bit/s/Hz.

In designing SOSTTC, the transmission matrices in an OSTBC, $W^{\left(1\right)}\left(0\right)$ or $W^{\left(1\right)}\left(\pi \right)$, are assigned to the branches that diverge from a state in the trellis. Therefore, any pair of coded paths in the trellis can achieve full diversity because they come from the same orthogonal code. Since the super-orthogonal code has an intra-set CGD of 0, full diversity cannot be achieved when the traditional MLC scheme utilizes the set partitioning of SOC. According to the rank and determinant criteria described in [24], the aim of the architecture is to maximize CGD and achieve full diversity over all pairs of distinct codewords.

In this research, we introduce a novel coding scheme called inter-multilevel super-orthogonal space–time coding (IM-SOSTC), which provides inter-level dependency between adjacent multilevel coded blocks and hence, guarantees full diversity. This proposed coding scheme was motivated by the multilevel coding structure in [41]. In this section, the proposed scheme is explained through examples and a general design rule is then provided.

**Figure 6:**
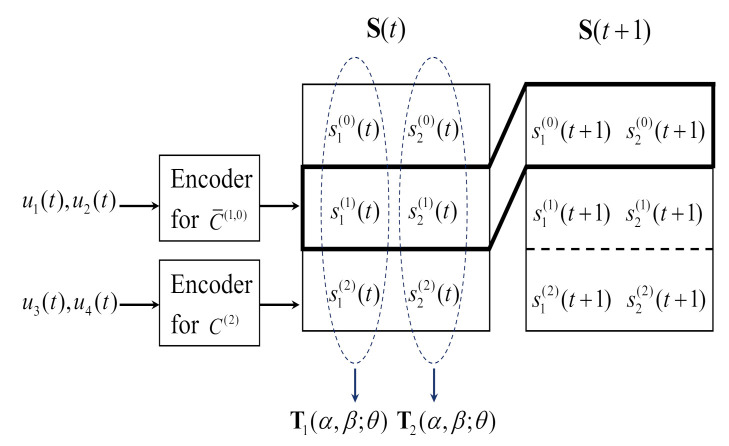
The encoding of IM-SOSTC-1.

**Example** **2.** *Considering the three-level partition chain P(3) that was constructed in Example 1, the encoding of the inter-multilevel super-orthogonal space–time code (IM-SOSTC-1) is presented in Figure 6.*

Let $S\left(t\right)=\left(\begin{array}{cc} s_{1}^{\left(0\right)}\left(t\right) & s_{2}^{\left(0\right)}\left(t\right)\\ s_{1}^{\left(1\right)}\left(t\right) & s_{2}^{\left(1\right)}\left(t\right)\\ s_{1}^{\left(2\right)}\left(t\right) & s_{2}^{\left(2\right)}\left(t\right) \end{array}\right)$ represent the coded block that is formed for the *t*th time segment, where $s_{1}^{\left(0\right)}\left(t\right)$ and $s_{2}^{\left(0\right)}\left(t\right)$ were determined in the previous time segment and $s_{1}^{\left(1\right)}\left(t\right),s_{2}^{\left(1\right)}\left(t\right),$$s_{1}^{\left(2\right)}\left(t\right)$, and $s_{2}^{\left(2\right)}\left(t\right)$ are determined in the current time segment. The encoding of the *t*th time segment includes two successive coded blocks, represented by (S(t) and S(t+1)). During the first level of encoding, $\left(s_{1}^{\left(1\right)}\left(t\right),s_{2}^{\left(1\right)}\left(t\right),s_{1}^{\left(0\right)}\left(t+1\right),s_{2}^{\left(0\right)}\left(t+1\right)\right)$ is a codeword for a (4, 2) binary linear code ${\bar{C}}^{\left(1,0\right)}$. Thus, ${\bar{C}}^{\left(1,0\right)}$ is a composition of ${\hat{C}}^{\left(1\right)}⊕C^{\left(1\right)}$ and $C^{\left(0\right)}$ and the generator matrix of ${\bar{C}}^{\left(1,0\right)}$ is $\left[\begin{array}{cc} {\hat{G}}^{\left(1\right)} & G^{\left(0\right)}\\ G^{\left(1\right)} & 0 \end{array}\right]=\left[\begin{array}{cc} 01 & 11\\ 11 & 00 \end{array}\right],$ where $G^{\left(1\right)}$ is the generator matrix of the (2, 1, 2) repetition code $C^{\left(1\right)},$$\left[\begin{array}{c} {\hat{G}}^{\left(1\right)}\\ G^{\left(1\right)} \end{array}\right]$ is the generator matrix of the (2, 2, 1) code ${\hat{C}}^{\left(1\right)}⊕C^{\left(1\right)},$ and $G^{\left(0\right)}$ is the generator matrix of the (2, 1, 2) repetition code $C^{\left(0\right)}$. During the second level of encoding, $\left(s_{1}^{\left(2\right)}\left(t\right),s_{2}^{\left(2\right)}\left(t\right)\right)$ is a codeword for the (2, 2, 1) universal code $C^{\left(2\right)}.$ For i=1,2, the column vector ${\left(s_{i}^{\left(0\right)}{\left(t\right)}_{}s_{i}^{\left(1\right)}{\left(t\right)}_{}s_{i}^{\left(2\right)}\left(t\right)\right)}^{T}$ is mapped onto the transmission matrix $T_{i}\left(\alpha ,\beta ;\theta \right).$ Four message bits $u_{1}\left(t\right),$$u_{2}\left(t\right),u_{3}\left(t\right),u_{4}\left(t\right)$ are encoded into two transmission matrices, which contain four BPSK symbols for the *t*th time segment. Therefore, the average spectral efficiency is 1 bit/s/Hz.

Now, the minimum CGD of IM-SOSTC-1 is calculated. Let $\left(u_{1}\left(t\right),u_{2}\left(t\right),u_{3}\left(t\right),u_{4}\left(t\right)\right)$ and $\left(u_{1}^{\prime }\left(t\right),u_{2}^{\prime }\left(t\right),u_{3}^{\prime }\left(t\right),u_{4}^{\prime }\left(t\right)\right)$ represent different message blocks and let (S(t),S(t+1)) and $\left(S^{\prime }\left(t\right),S^{\prime }\left(t+1\right)\right)$ represent the corresponding coded blocks. Note that the intra-set CGDs of $W^{\left(0\right)},W^{\left(1\right)},$ and $W^{\left(2\right)}$ can be calculated as: $\det\left(\begin{array}{cc} 4 & \;-4\\ \;-4 & 4 \end{array}\right)=0{,}_{}\det\left(\begin{array}{cc} 4 & 0\\ 0 & 4 \end{array}\right)=16,$ and $\det\left(\begin{array}{cc} 8 & 0\\ 0 & 8 \end{array}\right)=64,$ respectively.

Consider the following:

1. When $u_{1}\left(t\right)\neq u_{1}^{\prime }\left(t\right),$ it can be seen from the generator matrix of ${\bar{C}}^{\left(1,0\right)}$ that $S\left(t\right)\neq S^{\prime }\left(t\right)$ and $S\left(t+1\right)\neq S^{\prime }\left(t+1\right).$ Since the minimum Hamming distances of ${\hat{C}}^{\left(1\right)}⊕C^{\left(1\right)}$ and $C^{\left(0\right)}$ are 1 and 2, respectively, the minimum coding gain distance between (S(t),S(t+1)) and $\left(S^{\prime }\left(t\right),S^{\prime }\left(t+1\right)\right)$ can be computed as: $\det\left\{{}_{}\left(\begin{array}{cc} 4 & 0\\ 0 & 4 \end{array}\right)+\left(\begin{array}{cc} 4 & \;-4\\ \;-4 & 4 \end{array}\right)\left(\begin{array}{cc} 4 & \;-4\\ \;-4 & 4 \end{array}\right){}_{}\right\}=80.$
2. When $u_{1}\left(t\right)=u_{1}^{\prime }\left(t\right)$ and $u_{2}\left(t\right)\neq u_{2}^{\prime }\left(t\right),$ then $S\left(t\right)\neq S^{\prime }\left(t\right).$ Since the minimum Hamming distance of $C^{\left(1\right)}$ is 2, the minimum CGD between S(t) and $S^{\prime }\left(t\right)$ is calculated as:
  $\det\left\{{}_{}\left(\begin{array}{cc} 4 & 0\\ 0 & 4 \end{array}\right)+\left(\begin{array}{cc} 4 & 0\\ 0 & 4 \end{array}\right){}_{}\right\}=64$.
3. When $\left(u_{1}\left(t\right),u_{2}\left(t\right)\right)=\left(u_{1}^{\prime }\left(t\right),u_{2}^{\prime }\left(t\right)\right)$ and $\left(u_{3}\left(t\right),u_{4}\left(t\right)\right)\neq (u_{3}^{\prime }\left(t\right),$$u_{4}^{\prime }\left(t\right))$, then $S\left(t\right)\neq S^{\prime }\left(t\right).$ Since the minimum Hamming distance of $C^{\left(2\right)}$ is 1, the minimum CGD between S(t) and $S^{\prime }\left(t\right)$ is calculated as: $\det\left\{{}_{}\left(\begin{array}{cc} 8 & 0\\ 0 & 8 \end{array}\right){}_{}\right\}=64.$

**Figure 7:**
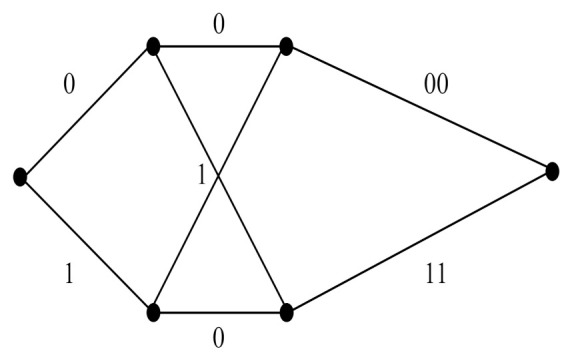
The trellis of ${\bar{C}}^{\left(1,0\right)}$ in IM-SOSTC-1.

From the above conditions, the minimum CGD of IM-SOSTC-1 is 64. The simple two-state trellis of ${\bar{C}}^{\left(1,0\right)}$ is shown in Figure 7, which combines two partial trellises for ${\hat{C}}^{\left(1\right)}⊕C^{\left(1\right)}$ and $C^{\left(0\right)}$.

**Figure 8:**
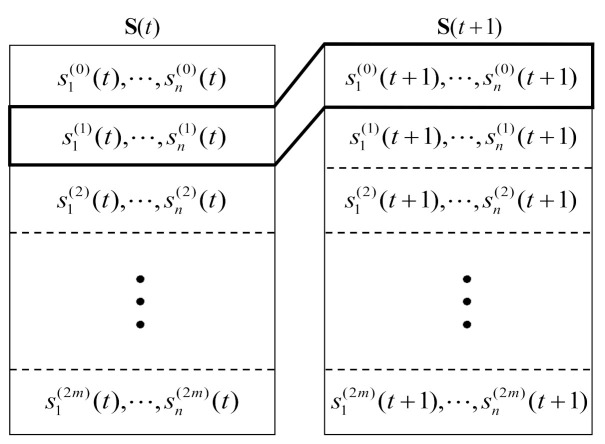
The coding configuration of IM-SOSTC.

Now, the systematic design method for IM-SOSTC is described. Let $W^{\left(1\right)}=W^{\left(1\right)}\left(0\right)=\left\{T(\alpha ,\beta ;0)\right\},$ where α and β are *M*-ary constellation symbols. If $M=2^{m},$ a 2*m*-level partitioning chain of $W^{\left(1\right)}/W^{\left(2\right)}/\cdots /W^{\left(2m\right)}$ with set partitioning can be constructed according to the CGD criterion introduced in [27]. By letting the super-orthogonal code $W^{\left(0\right)}$ denote the union of $W^{\left(1\right)}\left(0\right)$ and $W^{\left(1\right)}\left(\theta \right),$ a (2*m*+1)-level partitioning chain $P\left(2m+1\right)=W^{\left(0\right)}/W^{\left(1\right)}/W^{\left(2\right)}/\cdots /W^{\left(2m\right)}$ can be built. The coding configuration is presented in Figure 8. The coded block that is formed for the *t*th time segment is represented by $S\left(t\right)=\left(\begin{array}{ccc} s_{1}^{\left(0\right)}\left(t\right) & \cdots & s_{n}^{\left(0\right)}\left(t\right)\\ \vdots & \ddots & \vdots \\ s_{1}^{\left(2m\right)}\left(t\right) & \cdots & s_{n}^{\left(2m\right)}\left(t\right) \end{array}\right),$ where $s_{1}^{\left(0\right)}\left(t\right),\cdots ,s_{n}^{\left(0\right)}\left(t\right)$ were determined in a previous time segment. During the first level of the encoding, $\left(s_{1}^{\left(1\right)}\left(t\right),\cdots ,s_{n}^{\left(1\right)}\left(t\right),s_{1}^{\left(0\right)}\left(t+1\right),\cdots ,s_{n}^{\left(0\right)}\left(t+1\right)\right)$ is a codeword for the ${\bar{C}}^{\left(1,0\right)}$ of length 2*n*, which combines ${\hat{C}}^{\left(1\right)}⊕C^{\left(1\right)}$ and $C^{\left(0\right)}$.

The generator matrix of ${\bar{C}}^{\left(1,0\right)}$ is represented as: $\left[\begin{array}{cc} {\hat{G}}^{\left(1\right)} & G^{\left(0\right)}\\ G^{\left(1\right)} & 0 \end{array}\right].$ During the *p*th level of the encoding, p=2,3,⋯,2m,$\left(s_{1}^{\left(p\right)},s_{2}^{\left(p\right)},\cdots ,s_{n}^{\left(p\right)}\right)$ is designed to be a codeword for an $\left(n,k^{\left(p\right)},d^{\left(p\right)}\right)$ binary block code $C^{\left(p\right)}$ of length *n* with dimension $k^{\left(p\right)}$ and a minimum Hamming distance of $d^{\left(p\right)}$. For i=1,2,⋯,n, the column vector ${\left(s_{i}^{\left(0\right)}\left(t\right)\cdots s_{i}^{\left(2m\right)}\left(t\right)\right)}^{T}$ is mapped onto the transmission matrix $T_{i}\left(\alpha ,\beta ;\theta \right)$ according to the partition chain P(2m+1). The associated IM-SOSTC scheme is denoted as $\Omega \left({\bar{C}}^{\left(1,0\right)},C^{\left(2\right)},\cdots ,C^{\left(2m\right)};P\left(2m+1\right)\right)$.

Let $k^{\left(0\right)}$ and $k^{\left(1\right)}$ denote the dimensions of $C^{\left(0\right)}$ and $C^{\left(1\right)},$ respectively, and let the dimension of ${\bar{C}}^{\left(1,0\right)}$ be $k^{\left(0\right)}+k^{\left(1\right)}.$ As every coded block S(t) contains 2*n* signals, the average spectral efficiency is:

(4) $$\frac{\sum_{p=0}^{2m}k^{\left(p\right)}}{2n}bits/s/Hz.$$

The intra-set CGD of $W^{\left(0\right)}$ can be expressed as: $\det\left(\begin{array}{cc} \;\delta^{\left(0\right)} & -\delta^{\left(0\right)}\\ -\delta^{\left(0\right)} & \;\delta^{\left(0\right)} \end{array}\right)=0$. For p=1,2,⋯,2m, the intra-set CGD of $W^{\left(p\right)}$ is $\det\left(\begin{array}{cc} \delta^{\left(p\right)} & 0\\ 0 & \delta^{\left(p\right)} \end{array}\right)={\left(\delta^{\left(p\right)}\right)}^{2}=\Delta^{\left(p\right)}$. When $K=\sum_{p=0}^{2m}k^{\left(p\right)}$, let $\left(u_{1}\left(t\right),u_{2}\left(t\right),\cdots ,u_{K}\left(t\right)\right)$ and $\left(u_{1}^{\prime }\left(t\right),u_{2}^{\prime }\left(t\right),\cdots ,u_{K}^{\prime }\left(t\right)\right)$ represent two different message blocks and let (S(t),S(t+1)) and $\left(S^{\prime }\left(t\right),S^{\prime }\left(t+1\right)\right)$ represent the corresponding coded blocks. Consider the following conditions:

1. When $\left(u_{1}\left(t\right),u_{2}\left(t\right),\cdots ,u_{k_{0}}\left(t\right)\right)\neq \left(u_{1}^{\prime }\left(t\right),u_{2}^{\prime }\left(t\right),\cdots ,u_{k_{0}}^{\prime }\left(t\right)\right),$ then $S\left(t\right)\neq S^{\prime }\left(t\right)$ and $S\left(t+1\right)\neq S^{\prime }\left(t+1\right).$ Let ${\tilde{d}}^{\left(1\right)}$ and $d^{\left(0\right)}$ denote the minimum Hamming distances of ${\hat{C}}^{\left(1\right)}⊕C^{\left(1\right)}$ and $C^{\left(0\right)},$ respectively. The minimum coding gain distance between (S(t),S(t+1)) and $\left(S^{\prime }\left(t\right),S^{\prime }\left(t+1\right)\right)$ is expressed as:
  (5) $$\begin{array}{cc} \; & \det\left\{\sum_{i=1}^{{\tilde{d}}^{\left(1\right)}}\left(\begin{array}{cc} \delta^{\left(1\right)} & 0\\ 0 & \delta^{\left(1\right)} \end{array}\right)+\sum_{i=1}^{d^{\left(0\right)}}\left(\begin{array}{cc} \;\delta^{\left(0\right)} & -\delta^{\left(0\right)}\\ -\delta^{\left(0\right)} & \;\delta^{\left(0\right)} \end{array}\right)\right\}\\ \; & ={\left({\tilde{d}}^{\left(1\right)}\right)}^{2}\Delta^{\left(1\right)}+2{\tilde{d}}^{\left(1\right)}d^{\left(0\right)}\delta^{\left(1\right)}\delta^{\left(0\right)}. \end{array}$$
2. When $(u_{1}\left(t\right),u_{2}\left(t\right)$, ..., $u_{k_{0}}\left(t\right))$ = $(u_{1}^{\prime }\left(t\right),u_{2}^{\prime }\left(t\right)$, ..., $u_{k_{0}}^{\prime }\left(t\right))$ and $(u_{k_{0}+1}\left(t\right)$, $u_{k_{0}+2}\left(t\right)$, ..., $u_{k_{0}+k_{1}}\left(t\right))\neq (u_{k_{0}+1}^{\prime }\left(t\right)$, $u_{k_{0}+2}^{\prime }\left(t\right)$, ..., $u_{k_{0}+k_{1}}^{\prime }\left(t\right))$, then $S\left(t\right)\neq S^{\prime }\left(t\right)$. Let $d^{\left(1\right)}$ denote the minimum Hamming distance of $C^{\left(1\right)}$. The minimum CGD between S(t) and $S^{\prime }\left(t\right)$ can be expressed as:
  (6) $$\det\left\{{}_{}\sum_{i=1}^{d^{\left(1\right)}}\left(\begin{array}{cc} \delta^{\left(1\right)} & 0\\ 0 & \delta^{\left(1\right)} \end{array}\right)\right\}={\left(d^{\left(1\right)}\right)}^{2}\Delta^{\left(1\right)}.$$
3. When $(u_{1}\left(t\right),u_{2}\left(t\right)$, ..., $u_{k_{0}+k_{1}}\left(t\right))$ = $(u_{1}^{\prime }\left(t\right),u_{2}^{\prime }\left(t\right)$, ..., $u_{k_{0}+k_{1}}^{\prime }\left(t\right))$ and $(u_{k_{0}+k_{1}+1}\left(t\right)$, $u_{k_{0}+k_{1}+2}\left(t\right)$, ..., $u_{K}\left(t\right))\neq (u_{k_{0}+k_{1}+1}^{\prime }\left(t\right),u_{k_{0}+k_{1}+2}^{\prime }\left(t\right)$, ..., $u_{K}^{\prime }\left(t\right))$, then $S\left(t\right)\neq S^{\prime }\left(t\right)$. At the *p*th level, p=2,⋯,2m, the minimum CGD between S(t) and $S^{\prime }\left(t\right)$ is defined as:
  (7) $$\det\left\{{}_{}\sum_{i=1}^{d^{\left(p\right)}}\left(\begin{array}{cc} \delta^{\left(p\right)} & 0\\ 0 & \delta^{\left(p\right)} \end{array}\right)\right\}={\left(d^{\left(p\right)}\right)}^{2}\Delta^{\left(p\right)}.$$
  Based on the above conditions, the minimum CGD of $\Omega \left({\bar{C}}^{\left(1,0\right)},C^{\left(2\right)},\cdots ,C^{\left(2m\right)};P\left(2m+1\right)\right)$ is:
  (8) $$\;\min_{1\leq p\leq 2m}\left\{\begin{array}{cc} {\left({\tilde{d}}^{\left(1\right)}\right)}^{2}\Delta^{\left(1\right)}+2{\tilde{d}}^{\left(1\right)}d^{\left(0\right)}\delta^{\left(1\right)}\delta^{\left(0\right)}, & {\left(d^{\left(p\right)}\right)}^{2}\Delta^{\left(p\right)} \end{array}\right\}.$$

We adopted the suboptimal multistage decoder from [42] to keep the decoding complexity low. Moreover, the Viterbi soft decoding algorithm was computed on the trellises of component codes ${\bar{C}}^{\left(1,0\right)},C^{\left(2\right)},\cdots ,C^{\left(2m\right)}.$

**Example** **3.** *Considering the QPSK constellation, when $W^{\left(0\right)}=W^{\left(1\right)}\left(0\right)⋃W^{\left(1\right)}\left(\pi \right)$, a five-level partition chain $P\left(5\right)=W^{\left(0\right)}/W^{\left(1\right)}/\cdots /W^{\left(4\right)}$ can be constructed. It can be calculated that $\Delta^{\left(0\right)},\Delta^{\left(1\right)},\Delta^{\left(2\right)},\Delta^{\left(3\right)},$ and $\Delta^{\left(4\right)}$ are 0, 4, 16, 16, and 64, respectively. Let n=2, ${\bar{C}}^{\left(1,0\right)}$ be the (4, 2) code with the generator matrix $\left[\begin{array}{cc} 01 & 11\\ 11 & 00 \end{array}\right],$ and $C^{\left(2\right)},C^{\left(3\right)},C^{\left(4\right)}$ be the (2,2,1) codes. It can be calculated from (4) and (8) that the minimum CGD and spectral efficiency of $\Omega \left({\bar{C}}^{\left(1,0\right)},C^{\left(2\right)},C^{\left(3\right)},C^{\left(4\right)};P\left(5\right)\right)$, (IM-SOSTC-2) are 16 and 2 bits/s/Hz, respectively. However, the IM-SOSTC scheme can be designed with much larger minimum CGD values for the price of lower spectral efficiency.*

**Example** **4.** *Let n= 2, ${\bar{C}}^{\left(1,0\right)}$ be the (4,1) code with the generator matrix $\left[\begin{array}{cc} 11 & 11 \end{array}\right],$ let $C^{\left(2\right)},C^{\left(3\right)}$ be the (2, 1, 2) codes, and let $C^{\left(4\right)}$ be the (2, 2, 1) code. Note that $C^{\left(1\right)}$ is the (2, 0) code. It can be calculated from (4) and (8) that the minimum CGD and spectral efficiency of $\Omega \left({\bar{C}}^{\left(1,0\right)},C^{\left(2\right)},C^{\left(3\right)},C^{\left(4\right)};P\left(5\right)\right)$, (IM-SOSTC-3) are 64 and 1.25 bits/s/Hz, respectively. It is worth mentioning that the spectral efficiency can be increased when the length of the component codes is increased.*

**Figure 9:**
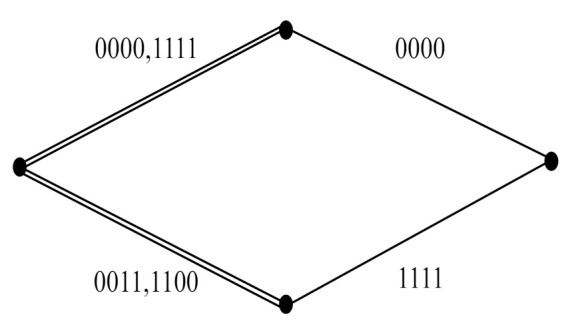
The trellis of ${\bar{C}}^{\left(1,0\right)}$ in IM-SOSTC-4.

**Example** **5.** *Let n= 4, ${\bar{C}}^{\left(1,0\right)}$ be the (8,2) code with the generator matrix $\left[\begin{array}{cc} 0011 & 1111\\ 1111 & 0000 \end{array}\right],$ let $C^{\left(2\right)},C^{\left(3\right)}$ be the (4, 3, 2) codes, and let $C^{\left(4\right)}$ be the (4, 4, 1) code. The minimum CGD and spectral efficiency of $\Omega \left({\bar{C}}^{\left(1,0\right)},C^{\left(2\right)},C^{\left(3\right)},C^{\left(4\right)};P\left(5\right)\right)$, (IM-SOSTC-4) are 64 and 1.5 bits/s/Hz, respectively. The two-state trellis of ${\bar{C}}^{\left(1,0\right)}$ is shown in Figure 9.*

## 4. Results and Performance Evaluation

**Figure 10:**
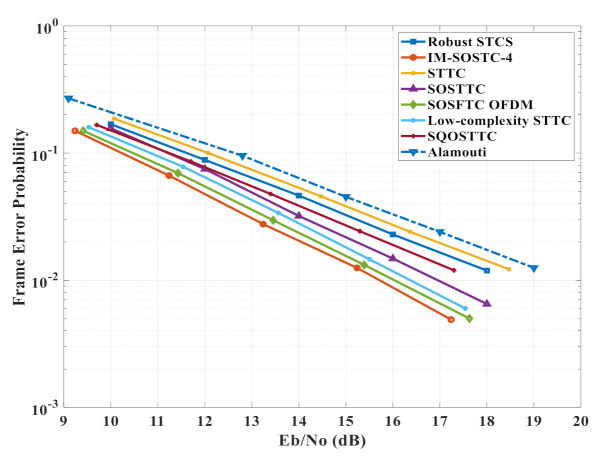
The simulation results of the two-state IM-SOSTC-4 vs. the other two-state coding schemes that use a QPSK constellation.

The simulation results for the frame error rate (FER) performance of the two-state IM-SOSTC-4 compared to other related low complexity orthogonal and super-orthogonal scheme designs are presented in Figure 10. In all simulations, two transmission antennas and one receiving antenna were considered on the quasi-static Rayleigh fading channel. Moreover, each segment consisted of 128 symbols out of each transmission antenna. It was also assumed that the path gains were constant over a frame of length *n* and varied from one frame to another. The simulation was performed using C language. From Figure 10, it can be noted that IM-SOSTC-4 surpassed the other related schemes in terms of coding gain. This coding gain achievement is reasonable because the minimum CGD of IM-SOSTC-4 is 64, which is considered high compared to the CGDs of other schemes. In general, the coding gain distance was high for low rate codes and hence, there was a trade-off between coding gain and the code rate. In addition, the super-orthogonal codes from [27,28,31] outperformed the orthogonal codes from [23,24,26,30], as can be observed from Figure 10.

It is worth mentioning that IM-SOSTC surpassed not only the other STBCs, but also STTCs. Additionally, the decoding complexity of the proposed IM-SOSTC is low because of the suboptimal multistage decoder and the squared Euclidean distance, which was used as the metric in the Viterbi soft decoding algorithm that was computed on the trellises of the component codes. Table 1 shows the main simulation parameters.

**Figure 11:**
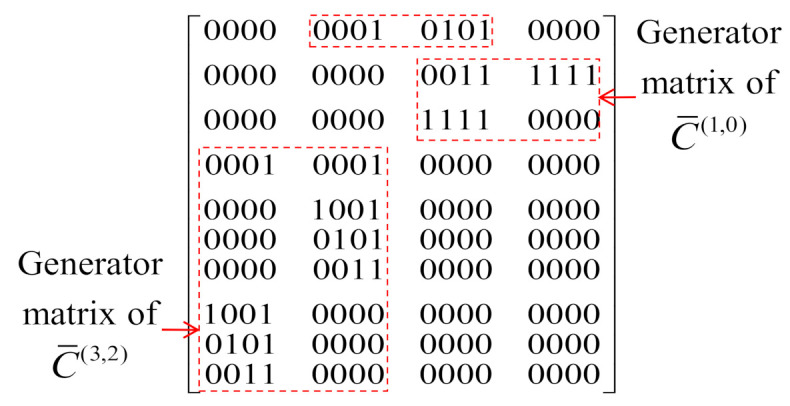
The generator matrix of ${\bar{C}}^{\left(3,2,1,0\right)}$.

**Figure 12:**
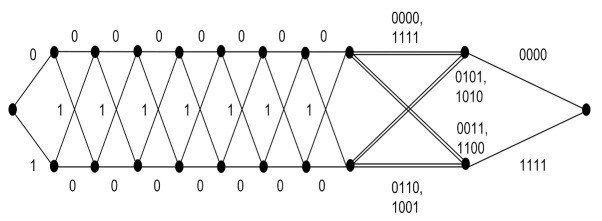
The trellis of ${\bar{C}}^{\left(3,2,1,0\right)}$.

Table 2 shows the coding gain improvement of the IM-SOSTC-4 over the popular Alamouti scheme compared to the other coding schemes. Note that the spectral efficiency could be further improved by adding more inter-level dependency. For example, in IM-SOSTC-4, we could form the composition of ${\tilde{C}}^{\left(3\right)}$ and ${\tilde{C}}^{\left(2\right)},$ denoted as ${\bar{C}}^{\left(3,2\right)},$ by using the squaring construction in which ${\tilde{C}}^{\left(p\right)}={\hat{C}}^{\left(p\right)}⊕C^{\left(p\right)}{,}_{}p=2,3.$ The generator matrix of ${\bar{C}}^{\left(3,2\right)}$ was $\left[\begin{array}{cc} {\hat{G}}^{\left(3\right)} & {\hat{G}}^{\left(2\right)}\\ 0 & G^{\left(2\right)}\\ G^{\left(3\right)} & 0 \end{array}\right],$ where $G^{\left(p\right)}$ and ${\hat{G}}^{\left(p\right)}$ are the generator matrices of $C^{\left(p\right)}$ and ${\hat{C}}^{\left(p\right)},$ respectively. The inter-level dependency provided one additional message bit. By repeating a similar concept, the inter-level dependency between ${\bar{C}}^{\left(3,2\right)}$ and ${\bar{C}}^{\left(1,0\right)}$ generated the composite code ${\bar{C}}^{\left(3,2,1,0\right)}$. The generator matrix and the two-state trellis of ${\bar{C}}^{\left(3,2,1,0\right)}$ are shown in Figure 11 and Figure 12, respectively. It could then be calculated that the minimum CGD of $\Omega \left({\bar{C}}^{\left(3,2,1,0\right)},C^{\left(4\right)};P\left(5\right)\right)$ was still 64 while spectral efficiency could be increased to 1.75 bits/s/Hz.

**Figure 13:**
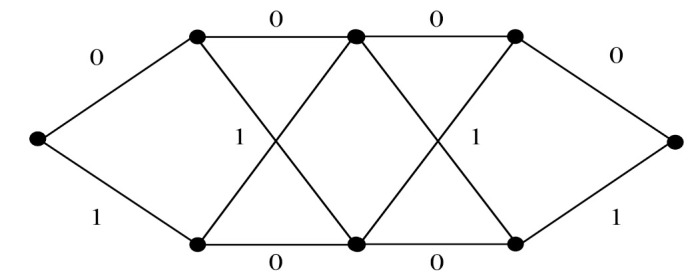
The trellis of $C^{\left(2\right)}$ and $C^{\left(3\right)}$, (4, 3, 2), in IM-SOSTC-4.

**Figure 14:**
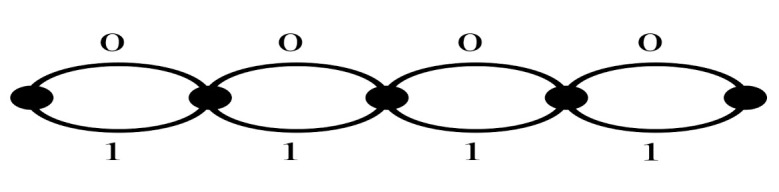
The trellis of $C^{\left(4\right)}$, (4, 4, 1), in IM-SOSTC-4.

Additionally, the trellis complexity of ${\bar{C}}^{\left(1,0\right)}$ in IM-SOSTC-4 was 27, in which the trellis complexity refers to 24 additions and 3 comparisons, as shown in Figure 9. $C^{\left(2\right)}$ and $C^{\left(3\right)}$ in IM-SOSTC-4 were the (4, 3, 2) code. In Figure 13, the number of additions was 12 and the number of comparisons was 5. Therefore, the trellis complexity of $C^{\left(2\right)}$ and $C^{\left(3\right)}$ was 17. Moreover, the trellis complexity of $C^{\left(4\right)}$ in IM-SOSTC-4 was 12, in which the number of additions was 8 and the number of comparisons was 4, as shown in Figure 14. Hence, the total trellis complexity was 73. Nevertheless, the total trellis complexity could be normalized and divided by eight because each coded block contained eight QPSK signals; hence, the average trellis complexity was only 73/8 = 9.125 operations per two-dimensional signal.

## 5. Conclusions

In this paper, an original low-cost coding structure was proposed, namely inter-multilevel super-orthogonal space–time coding (IM-SOSTC). This flexible coding design allows for inter-level dependency between adjacent multilevel coded blocks to improve coding gain and guarantee full diversity whilst maintaining reasonable spectral efficiency in a wireless fading and interfering channel. Hence, the proposed IM-SOSTC can meet the reliability requirements of ZigBee-based IoMT communications while achieving low complexity. The IM-SOSTC was compared to the other related schemes of STTC, Robust STCS, SOSTTC, SQOSTTC, low complexity STTC, and SOSFTC OFDM over Alamouti and the IM-SOSTC showed significant high coding gains reaching 3.97 dB, while the other schemes reached 0.56 dB, 1.16 dB, 2.73 dB, 1.82 dB, 3.29 dB, and 3.67 dB at an FEP of $10^{-2}$, respectively. Moreover, the average trellis complexity was only 9.125 operations per two-dimensional signal. The data rate can be raised by increasing the amount of inter-level dependency. However, the increase in the amount of inter-level dependency affects the coding gain and reliability. Future work will focus on the study of the maximum spectral efficiency for a given coding gain distance (CGD) and an analysis of the trade-off between complexity and reliability.

## Figures and Tables

**Table 1:** A summary of the simulation parameters.

Parameters	Configurations
Frame Length	128 symbols
States	2
Modulator	QPSK
Number of MIMO Antennas	2×1
Channel	Quasi-Static Rayleigh Fading

**Table 2:** The coding gain improvements over Alamouti.

Schemes/FEP	$10^{-1}$	$10^{-2}$
STTC [24]	0.56 dB	0.56 dB
Robust STCS [26]	1.16 dB	1.16 dB
SOSTTC [27]	1.52 dB	2.73 dB
SQOSTTC [28]	1.52 dB	1.82 dB
Low Complexity STTC [30]	1.97 dB	3.29 dB
SOSFTC OFDM [31]	2.28 dB	3.67 dB
**IM-SOSTC-4**	**2.58 dB**	**3.97 dB**

## Data Availability

Not applicable.
